# The influence of college students’ AI literacy on perceived usefulness: a moderated mediation model with AI anxiety and trust across disciplinary backgrounds

**DOI:** 10.3389/fpsyg.2026.1731653

**Published:** 2026-09-01

**Authors:** Zihan Li, Jiaying Han, Liwei Wu, Xiaogang Wang

**Affiliations:** 1 Southwest Minzu University, Chengdu, China; 2 Key Research Institute of Humanities and Social Sciences in Southwest Minzu University, State Ethnic Affairs Commission, Chengdu, China

**Keywords:** AI anxiety, AI literacy, college students, disciplinary differences, perceived usefulness, trust

## Abstract

**Background:**

To investigate how AI literacy influences perceived usefulness among university students from different disciplinary backgrounds, this study examines the roles of AI anxiety and trust.

**Methods:**

Based on 563 valid samples collected from university students, a moderated mediation model was utilized to explore AI anxiety as a mediator and trust as a moderator across various disciplinary backgrounds.

**Results:**

Results indicate that: (1) AI literacy significantly and positively predicted perceived usefulness, whereas AI anxiety negatively predicted perceived usefulness; (2) AI anxiety mediated the relationship between AI literacy and perceived usefulness; (3) Trust significantly moderated the relationship between AI literacy and AI anxiety, with high levels of trust buffering anxiety arising from low AI literacy; (4) Students from different disciplinary backgrounds exhibited differences in AI literacy, AI anxiety, trust, and perceived usefulness, yet trust’s moderating effect remained consistent across disciplines.

**Discussion:**

This study extends the Technology Acceptance Model (TAM) within an AI context, revealing the synergistic mechanisms of cognitive, affective, and socio-psychological factors in university students’ AI adoption processes. The findings provide actionable guidance for AI education in higher education by emphasizing the need for discipline-specific curricula alongside targeted interventions that enhance trust and reduce AI anxiety, thereby improving students’ perceived usefulness and engagement with AI technologies.

## Introduction

Artificial intelligence =(AI) is reshaping the educational landscape and progressively reshaping college students’ learning styles, pathways of knowledge acquisition, and problem-solving patterns. However, whether AI technologies can truly be accepted by college students does not depend solely on their objective technological advantages; rather, it largely depends on individuals’ willingness to psychologically and behaviorally embrace and use these technologies (Shin, 2021; Choung et al., 2023).

In explaining users’ technology adoption behavior, the Technology Acceptance Model (TAM) provides an important theoretical framework, proposing that perceived usefulness is a key antecedent influencing technology acceptance (Davis, 1989). Traditional TAM is primarily grounded in the assumption of “rational cognition,” emphasizing technology evaluation based on improvements in individual efficiency and performance, while overlooking the complex emotional responses and socio-psychological resources involved in AI-related contexts. Compared with traditional information technologies, AI is characterized by greater autonomy, opacity, and unpredictability (Shin, 2020). When interacting with AI, users not only evaluate “whether it is useful,” but also worry about “whether it is reliable,” “whether it will replace them,” and “whether they will lose control.” Therefore, the acceptance of AI technologies has moved beyond the purely rational decision-making logic proposed in traditional TAM, evolving instead into a dynamic process jointly shaped by cognitive, emotional, and socio-psychological factors (Cheng et al., 2022; Siau and Wang, 2018).

Against this background, explaining college students’ perceived usefulness of AI solely from a cognitive perspective is no longer sufficient; research needs to further incorporate emotional variables and socio-psychological resource variables. Among these, AI anxiety refers to the feelings of unease, worry, and perceived threat that individuals experience when confronting AI technologies (Johnson and Verdicchio, 2017). Previous studies have shown that AI anxiety significantly reduces individuals’ attitudes toward technology acceptance and their intention to use AI technologies (Wang and Wang, 2022). Meanwhile, due to the “black-box” nature of AI, users are often unable to fully understand its decision-making logic; consequently, trust has become a critical psychological resource influencing AI acceptance (Shin, 2021). High levels of trust can reduce perceptions of risk and uncertainty, thereby buffering the negative impact of AI anxiety on technology acceptance (Gefen et al., 2003). Furthermore, the development of both AI anxiety and AI trust is inseparable from individuals’ AI literacy. AI literacy encompasses not only an understanding of AI principles, application methods, and ethical risks, but also individuals’ ability to critically evaluate and responsibly use AI technologies (Long and Magerko, 2020; Wang et al., 2023). Individuals with higher levels of AI literacy tend to possess a stronger sense of technological controllability, are more likely to develop positive perceptions of AI, and experience lower levels of AI anxiety. Therefore, AI literacy may not only directly influence perceived usefulness, but may also indirectly affect college students’ acceptance of AI through AI anxiety.

In addition, a substantial body of research has indicated that students from different disciplinary backgrounds differ significantly in terms of knowledge structures, modes of thinking, and experiences with technology exposure. Students in the natural sciences (e.g., STEM and applied sciences) tend to place greater emphasis on the practicality and efficiency of technology, whereas students in the humanities and social sciences are more concerned with the ethical, cultural, and societal implications of AI (Elshaer et al., 2024; Qu et al., 2024; Raman et al., 2024; Stöhr et al., 2024; Thongsri et al., 2020). These disciplinary orientations may influence the relationships among AI literacy, AI anxiety, trust, and perceived usefulness. Therefore, disciplinary background may constitute an important grouping variable within the TAM framework.

### Perceived usefulness

Perceived usefulness refers to the degree to which an individual subjectively believes a particular technology can enhance their work or learning performance (Davis, 1989), and is considered one of the antecedent variables of technology adoption intention. In AI contexts, perceived usefulness reflects whether users believe AI can assist them in accomplishing tasks more efficiently, optimizing decision-making, or enhancing experiences (Choung et al., 2023). Existing research has validated the crucial role of perceived usefulness in influencing usage intention across diverse domains, including online shopping (Gefen et al., 2003) and mobile banking (Luarn and Lin, 2005; Venkatesh and Davis, 2000; Venkatesh et al., 2003). However, the opacity and uncertainty inherent in AI technology (Shin, 2020) complicate its evaluation process. Users no longer perceive it merely as a passive tool but engage with an “agent” possessing a degree of autonomy, assessing the value of its outputs (Cheng et al., 2022; Hoffman et al., 2013). Consequently, this study posits that users’ perceived usefulness of AI technologies does not arise in isolation. It is profoundly influenced by users’ cognitive reserves (AI literacy) and emotional states (AI anxiety). Clarifying this pathway holds core guiding significance for designing AI systems that are more readily accepted by users.

### AI literacy

AI literacy constitutes the core cognitive competency required of individuals in the AI era (Ng, 2012). It encompasses not only the ability to understand, use, and evaluate AI technologies, but also the capacity for critical awareness of algorithmic mechanisms, ethical risks, and the broader societal implications of AI (Long and Magerko, 2020; Wang et al., 2023). It should be noted that AI literacy is not equivalent to general digital literacy or technological proficiency. Digital literacy primarily emphasizes information acquisition and the ability to use digital tools, whereas AI literacy further involves understanding and reflecting on algorithmic logic, model limitations, algorithmic bias, and privacy risks (Ng et al., 2021). Therefore, AI literacy reflects a deeper level of cognitive judgment rather than merely AI exposure or frequency of use.

Within the framework of the TAM, traditional perceived usefulness mainly emphasizes the extent to which technology enhances efficiency and performance (Venkatesh and Davis, 2000). In contrast, AI literacy influences not only individuals’ understanding of the functionality of AI tools, but also their comprehensive evaluations of AI reliability, risk, and value, thereby extending the traditional TAM explanatory framework centered on “instrumental efficiency.” Existing studies have shown that individuals with higher levels of AI literacy are not only more capable of effectively utilizing AI tools, but are also better able to identify algorithmic bias and critically reflect on the limitations of technology (Ng et al., 2021). Moreover, higher AI literacy implies a stronger understanding of AI operational logic and a greater sense of controllability, enabling individuals to more readily recognize the potential value of AI tools in the learning process and develop more positive perceptions of usefulness (Chiu et al., 2022).

Accordingly, the present study argues that AI literacy is not only a key cognitive antecedent of college students’ perceived usefulness, but also an important determinant of AI technology acceptance.

### The mediating role of AI anxiety

The rapid advancement of AI has brought uncertainties and risks that have triggered anxiety among individuals (Wang and Wang, 2022). With the proliferation of generative AI and intelligent learning systems, this anxiety has shown a marked upward trend. AI may lead to increasing concerns such as unemployment, privacy breaches, “black box” issues, and information transparency, resulting in a corresponding rise in public apprehension toward AI (Li and Zhao, 2025). Johnson and Verdicchio (2017) define AI anxiety as “the fear and concern of losing control over artificial intelligence.” Its origins are multidimensional, primarily encompassing four aspects: firstly, learning anxiety; secondly, job displacement anxiety; thirdly, socio-technological blindness anxiety; and fourthly, AI configuration anxiety (Wang et al., 2023).

According to Cognitive-Affective System Theory (Mayer and Salovey, 1995), an individual’s cognitive abilities influence their emotional responses, which in turn shape attitudes and behaviors. Wang and Wang (2022) conceptualize AI anxiety as a belief serving as a antecedent or mediator of behavioral intentions, linking causal factors and attitudes to subsequent actions. Existing research indicates that heightened AI anxiety significantly diminishes individuals’ willingness to adopt and utilized new technologies (Wang and Wang, 2022). Consequently, this study posits that individuals with high AI literacy exhibit lower AI anxiety due to clearer cognitive understanding and perceived control over technology. Conversely, those with low AI literacy, unable to comprehend or predict AI behavior, experience heightened uncertainty and anxiety, thereby diminishing perceived usefulness of AI. This implies AI anxiety mediates the pathway from “AI literacy → perceived usefulness.”

### The moderating role of trust

At the socio-psychological level, trust finds extensive application across multiple disciplines including psychology, sociology, economics, and human-computer interaction (Lee and See, 2004; Mayer et al., 1995; Xie and Zhou, 2025). Within the Technology Acceptance Model (TAM) framework, trust has also been demonstrated as a significant antecedent influencing users’ adoption of emerging technologies (Wu et al., 2011). An individual’s level of trust in AI significantly influences their attitudes and behaviors toward AI in learning and work contexts (McKnight et al., 2011; Basu and Singhal, 2016). Trust is regarded as a crucial psychological buffering mechanism, mitigating the negative impact of factors such as uncertainty and risk perception on user decision-making and attitudes (Mayer et al., 1995; Lee and See, 2004). Within technology acceptance contexts, high levels of trust assist users in overcoming concerns regarding AI’s “black box” characteristics, reducing perceived risks and thereby increasing their propensity to recognize AI’s usefulness (Gefen et al., 2003; Shin, 2021). Within China’s higher education environment, trust manifests not only as recognition of AI technology’s reliability but also as institutional trust in the educational system, information sources, and AI platforms. This constitutes an important cultural foundation underlying college students’ acceptance attitudes toward the perceived usefulness of AI. Trust thus transcends an individual psychological state, becoming a bridge connecting AI technology characteristics with educational context demands. This alignment renders the pathway “AI literacy → AI anxiety → perceived usefulness” more consistent with university students’ technology acceptance logic.

Consequently, this study posits that trust moderates the “AI literacy → AI anxiety” pathway: elevated trust levels mitigate anxiety stemming from low AI literacy, thereby stabilizing individuals’ positive perceptions and attitudes toward AI.

**FIGURE 1:**
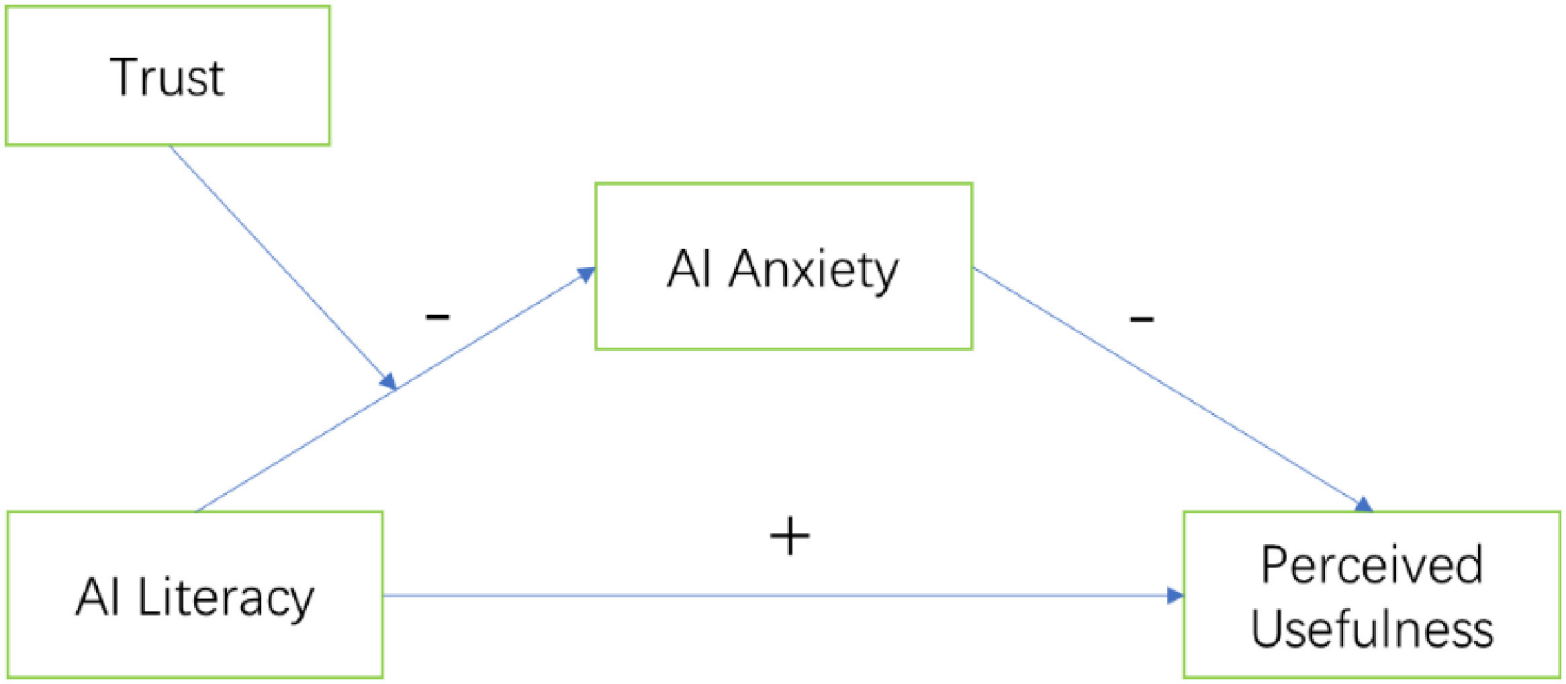
Research hypothesis model. AI literacy indirectly influences perceived usefulness through AI anxiety, while trust moderates the relationship between AI literacy and AI anxiety. Solid lines represent the hypothesized pathways; “+” indicates a positive relationship, whereas “−” indicates a negative relationship. This study employed PROCESS Model 7 to test the moderated mediation effect.

In summary, grounded in the Technology Acceptance Model (TAM), this study integrates a cognitive–emotional framework to examine the formation mechanism of college students’ perceived usefulness of AI from three dimensions: cognitive resources (AI literacy), emotional responses (AI anxiety), and socio-psychological resources (trust). Specifically, this study constructs and tests a moderated mediation model (see Figure 1) to investigate the mediating role of AI anxiety in the relationship between AI literacy and perceived usefulness, as well as the moderating role of trust in the pathway from AI literacy to AI anxiety. Furthermore, this study compares the stability of the model across different disciplinary backgrounds in order to examine whether this psychological mechanism demonstrates cross-disciplinary consistency.

Compared with previous studies that primarily focused on technological characteristics or single psychological variables, this study seeks to explain the formation process of college students’ perceived usefulness of AI from an integrated perspective encompassing cognitive, emotional, and socio-psychological resources. In doing so, this study not only extends the application boundary of TAM within intelligent education contexts, but also provides a new theoretical perspective for understanding the psychological mechanisms underlying human–AI interaction among college students in higher education settings.

Based on this theoretical foundation, the following hypotheses are proposed:

*H1:* AI literacy positively predicts college students’ perceived usefulness of AI;

*H2:* AI anxiety negatively predicts perceived usefulness;

*H3:* AI anxiety mediates the relationship between AI literacy and perceived usefulness;

*H4:* Trust moderates the relationship between AI literacy and AI anxiety, such that higher trust levels strengthen the negative predictive effect of AI literacy on AI anxiety.

## Materials and methods

### Subjects

This study employed convenience sampling and collected data through an online anonymous questionnaire. The questionnaire was distributed via the *Wenjuanxing* platform and targeted full-time undergraduate students. Participants were recruited through university course groups, student social media groups, and online campus communities. Eligible participants were required to be full-time undergraduate students enrolled in Chinese higher education institutions and to have basic experience with AI tools through prior exposure or use. Before accessing the formal questionnaire, participants were required to read an electronic informed consent statement and confirm their voluntary participation. All questionnaires were submitted anonymously, and no personally identifiable information was collected during the research process. The operational definitions of disciplinary backgrounds referenced the Catalog of Undergraduate Majors in Chinese Higher Education Institutions (2022 Edition), categorizing disciplines into two types based on knowledge system characteristics and methodological differences: Natural Sciences disciplines (encompassing science, engineering, agriculture, and medicine, with a core focus on investigating the objective laws of natural phenomena, primarily employing empirical research methods); Humanities and Social Sciences disciplines (encompassing Philosophy, Economics, Law, Education, Literature, History, Management, and Arts, emphasizing the interpretation of human society and cultural phenomena through a blend of critical and empirical research methods). A total of 600 questionnaires were collected over a 6-month data collection period. Following rigorous screening, 37 invalid questionnaires were excluded due to excessively short completion times or demonstrably patterned responses (e.g., selecting the same option for all questions). This yielded 563 valid questionnaires (exceeding the sample size requirement per Kendall’s empirical rule), representing an effective recovery rate of 93.8%. Among these, 303 participants (53.8%) were from humanities and social sciences disciplines, with an average age of 21.31 ± 1.56 years; 260 participants (46.2%) were from natural sciences disciplines, with an average age of 21.12 ± 1.36 years. Males constituted 33.9% of the sample, while females accounted for 66.1%. To reduce the potential influence of demographic variables on the relationships within the model, gender was controlled for in all subsequent PROCESS analyses conducted in this study.

### Measurement tools

#### Artificial intelligence literacy scale

The Artificial Intelligence Literacy Scale developed by Wang et al. (2023) was employed to measure university students’ AI literacy levels. This scale comprises 12 items across four dimensions: Awareness, Usage, Evaluation, and Ethics, with each dimension containing three items. In this study, the scale demonstrated an internal consistency coefficient of 0.72 among university student participants, indicating acceptable reliability.

#### Artificial intelligence anxiety scale

The measurement of AI anxiety employed the Artificial Intelligence Anxiety Scale developed by Wang and Wang (2022). This scale comprises 21 items across four dimensions: learning anxiety (8 items), job displacement anxiety (6 items), socio-technological illiteracy anxiety (4 items), and AI configuration anxiety (3 items). The scale employs a five-point Likert scoring system, with higher total scores indicating greater anxiety levels regarding AI technology. The internal consistency coefficient for this scale in the present study was 0.93, demonstrating excellent reliability.

#### Trust in AI scale

The Trust in AI Scale developed by Choung et al. (2023) was employed to measure university students’ trust levels in AI technology. This scale comprises 11 items across two dimensions: Human-like Trust and Functionality Trust. The scale employs a five-point Likert scoring system, with higher total scores indicating greater trust in AI technology. In this study, the scale demonstrated good internal consistency, with a coefficient of 0.86.

#### Perceived usefulness

The perceived usefulness of AI technology among university students was measured using 10 items from the perceived usefulness dimension of the classic scale developed by Davis (1989). The scale employed a five-point Likert scoring system, with higher total scores indicating stronger perceived usefulness of AI technology among the student participants. In this study, the scale demonstrated good internal consistency, with a coefficient of 0.80.

#### Data analysis and research design

Data analysis for this study employed SPSS 26.0 to conduct descriptive statistics, independent samples *t*-tests, and correlation analyses. Second, the PROCESS macro program was employed to analyze the moderated mediation model. Specifically, PROCESS Model 4 was used to test the mediation effect, whereas PROCESS Model 7 was applied to test the moderated mediation effect. All indirect effects were tested for significance using the bias-corrected Bootstrap method (5,000 repeated samples), with a 95% confidence interval (CI) not containing 0 as the significance criterion.

#### Common method bias test

Since all the variables in this study were collected through self-reported questionnaires, common method bias may exist. To assess the potential influence of common method bias, Harman’s single-factor test was conducted. All measurement items were subjected to an unrotated exploratory factor analysis (EFA). The results showed that nine factors with eigenvalues exceeding 1. The single factor with the highest variance explained accounted for only 27.15%, below the critical threshold of 40% (Podsakoff et al., 2003). Furthermore, all item loadings ranged between 0.30 and 0.77, with no single item exhibiting an abnormally high loading (e.g., approaching or exceeding 0.90). Therefore, it can be assumed that the data of the present study do not suffer from a serious problem of common method bias.

#### Multicollinearity test (VIF)

Before conducting the regression and PROCESS analyses, this study further examined multicollinearity among the major variables. The results showed that all tolerance values were above 0.20 (0.53–0.62), and all Variance Inflation Factor (VIF) values were below 10 (1.61–1.88), indicating that no serious multicollinearity problem existed in the model.

#### Ethics approval

This study was strictly followed the Declaration of Helsinki and approved by the Professor and Ethics Committee, School of Education and Psychology, Southwest Minzu University (Approval No. JX20251010). In this study, we obtained informed consent from the participants who participated in the questionnaire survey.

## Results

### Descriptive statistics and correlation analysis

Correlation analyses were conducted on the total scores of AI literacy, AI anxiety, trust, and perceived usefulness across the two disciplinary groups. The results indicated that significant correlations existed among all core variables in both groups (see Tables 1, 2).

**TABLE 1:** Correlations among the study variables (humanities and social sciences).

Variables	*M*	*SD*	AI literacy	AI anxiety	Trust	Perceived usefulness
AI literacy	49.14	4.35	1	1	1	1
AI anxiety	54.97	14.44	–0.49**			
Trust	43.85	5.46	0.60**	–0.57**		
Perceived usefulness	41.76	4.33	0.64**	–0.49**	0.74**	

*p < 0.05, **p < 0.01, ***p < 0.001.

**TABLE 2:** Correlations among the study variables (natural sciences).

Variables	*M*	*SD*	AI literacy	AI anxiety	Trust	Perceived usefulness
AI literacy	50.32	4.24	1	1	1	1
AI anxiety	49.90	15.98	–0.50**			
Trust	44.79	5.17	0.59**	–0.60**		
Perceived usefulness	42.53	3.41	0.63**	–0.55**	0.70**	

Sample size *N* = 563; M = Mean, SD = Standard Deviation. **p < 0.01 (two-tailed), same applies below.

Specifically, among students in the natural sciences, AI literacy was significantly and positively correlated with trust (*r* = 0.59, *p* < 0.01) and perceived usefulness (*r* = 0.63, *p* < 0.01), while being significantly and negatively correlated with AI anxiety (*r* = −0.50, *p* < 0.01). AI anxiety was significantly and negatively correlated with both trust (*r* = −0.60, *p* < 0.01) and perceived usefulness (*r* = −0.55, *p* < 0.01). In addition, trust was highly and positively correlated with perceived usefulness (*r* = 0.70, *p* < 0.01).

Among students in the humanities and social sciences, AI literacy was likewise significantly and positively correlated with trust (*r* = 0.60, *p* < 0.01) and perceived usefulness (*r* = 0.64, *p* < 0.01), while showing a significant negative correlation with AI anxiety (*r* = −0.49, *p* < 0.01). Furthermore, AI anxiety was significantly and negatively correlated with both trust (*r* = −0.57, *p* < 0.01) and perceived usefulness (*r* = −0.49, *p* < 0.01). Trust was also significantly and positively correlated with perceived usefulness (*r* = 0.74, *p* < 0.01).

### Group differences by disciplinary background (independent samples *t*-test)

To further investigate differences in core research variables among university students from distinct disciplinary backgrounds, this study employed an independent samples *t*-test to compare scores between humanities and social sciences (HSS) and natural sciences (NS) groups. As shown in Table 3, significant differences emerged between the two groups in AI anxiety, perceived usefulness, AI literacy, and trust. Humanities and social sciences students exhibited significantly higher levels of AI anxiety than their natural sciences counterparts. Conversely, natural sciences students scored significantly higher in perceived usefulness. The findings indicate that natural sciences students generally demonstrated higher AI literacy, trust, and perceived usefulness, whereas humanities and social sciences students exhibited greater levels of AI anxiety.

**TABLE 3:** Independent samples *t*-tests of the study variables.

Variables	Humanities and social sciences *(N = 303) M ± SD*	Natural sciences *(N = 260) M ± SD*	*t*	*p*
AI anxiety	54.97 ± 14.44	49.90 ± 15.98	3.96***	0.000
Perceived usefulness	41.76 ± 4.32	42.52 ± 3.41	–2.29*	0.022
AI literacy	49.14 ± 4.35	50.32 ± 4.24	–3.25**	0.001
Trust	43.85 ± 5.46	44.79 ± 5.16	–2.08*	0.038

*p < 0.05, **p < 0.01, ***p < 0.001.

### The relationship between AI literacy and perceived usefulness: a moderated mediation model test

Based on the testing method proposed by Hayes (2013) and Wen and Ye (2014), this study examines the relationship between AI literacy and perceived usefulness, the mediating effect of AI anxiety on this relationship, and the moderating effect of trust on the first half of this mediating pathway. First, using Model 4 of the SPSS PROCESS macro, bootstrap mediation analyses were conducted on the total sample (*N* = 563) after controlling for gender variables. The results are presented in Tables 4, 5.

**TABLE 4:** Mediation effect test table.

Outcome variable	Predictor variable	*B*	*SE*	*t*	*R* ^2^	*F*
AI anxiety	AI literacy	–1.72	0.13	–13.62***	0.30	117.45***
Perceived usefulness	AI literacy	0.57	0.03	19.34***	0.41	198.03***
Perceived usefulness	AI literacy	0.46	0.03	14.08***	0.46	156.93***
	AI anxiety	–0.06	0.010	–6.65***		

B, unstandardized coefficient; SE, standard error; *p < 0.05, **p < 0.01, ***p < 0.001.

**TABLE 5:** Table of mediated effects.

Variable path	Effect size	*SE*	95% CI		Effect (%)
			*LLCL*	*ULCL*	
Direct effect	0.46	0.03	0.40	0.53	80.94%
Indirect effects	0.11	0.02	0.07	0.15	19.06%
Total effect	0.57	0.03	0.51	0.63	100%

The results showed that AI literacy significantly and positively predicted perceived usefulness, with a significant total effect [*B* = 0.57, *SE* = 0.03, *t* = 19.34, *p* < 0.001, 95% CI (0.51, 0.63)]. After including AI anxiety as a mediating variable, AI literacy significantly and negatively predicted AI anxiety (*B* = −1.72, *SE* = 0.13, *t* = −13.62, *p* < 0.001), indicating that higher levels of AI literacy were associated with lower levels of AI anxiety. At the same time, AI anxiety significantly and negatively predicted perceived usefulness (*B* = −0.06, *SE* = 0.01, *t* = −6.65, *p* < 0.001), suggesting that higher AI anxiety was associated with lower perceived usefulness of AI.

After including for AI anxiety, the direct effect of AI literacy on perceived usefulness remained significant [*B* = 0.46, *SE* = 0.03, *t* = 14.08, *p* < 0.001, 95% CI (0.40, 0.53)], indicating that AI anxiety played a partial mediating role in the relationship between AI literacy and perceived usefulness. Furthermore, the bias-corrected Bootstrap results showed that the indirect effect was 0.11 [*BootSE* = 0.02, 95% CI (0.07, 0.15)], with the confidence interval excluding zero, thereby confirming the significance of the mediation effect. The indirect effect accounted for 19.06% of the total effect, whereas the direct effect accounted for 80.94% of the total effect.

Next, to examine the moderating role of trust in the relationship between AI literacy and AI anxiety, this study employed the PROCESS macro PROCESS Model 7 to conduct a moderated mediation analysis, with gender controlled for in the model. The results are presented in Table 6.

**TABLE 6:** Results of the moderated mediation regression analysis.

Variable	AI anxiety		Perceived usefulness	
	*B*	*t*	*B*	*t*
Constant	44.78	24.67***	46.17	77.47***
AI literacy	–0.97	–6.79***	0.46	14.08***
Trust	–1.29	–11.12***		
AI literacy* Trust	–0.109	–5.366***		
Gender	5.64	5.42***	–0.43	–1.61
AI anxiety			–0.06	–6.65***
Sample size	563		563	
*R* ^2^	0.43		0.46	
Adjusted *R*^2^	0.43		0.45	
*F*	108.23		156.93	

*p < 0.05, **p < 0.01, ***p < 0.001.

The results indicated that AI literacy significantly and negatively predicted AI anxiety (*B* = −0.97, *t* = −6.79, *p* < 0.001), suggesting that higher levels of AI literacy were associated with lower levels of AI anxiety. Trust also significantly and negatively predicted AI anxiety (*B* = −1.29, *t* = −11.12, *p* < 0.001). In addition, the interaction term between AI literacy and trust significantly predicted AI anxiety (*B* = −0.11, *t* = −5.37, *p* < 0.001), indicating that trust significantly moderated the relationship between AI literacy and AI anxiety.

In the regression model predicting perceived usefulness, AI literacy significantly and positively predicted perceived usefulness (*B* = 0.46, *t* = 14.08, *p* < 0.001), whereas AI anxiety significantly and negatively predicted perceived usefulness (*B* = −0.06, *t* = −6.65, *p* < 0.001). The overall explanatory power of the models was relatively high, with an *R*^2^ of 0.43 for the AI anxiety model and an *R*^2^ of 0.46 for the perceived usefulness model.

Taken together, these findings indicate that trust moderated the first stage of the mediating process through which AI literacy influenced perceived usefulness via AI anxiety. In other words, the effect of AI literacy on AI anxiety varied according to different levels of trust, thereby supporting the moderated mediation model (Wen and Ye, 2014).

**FIGURE 2:**
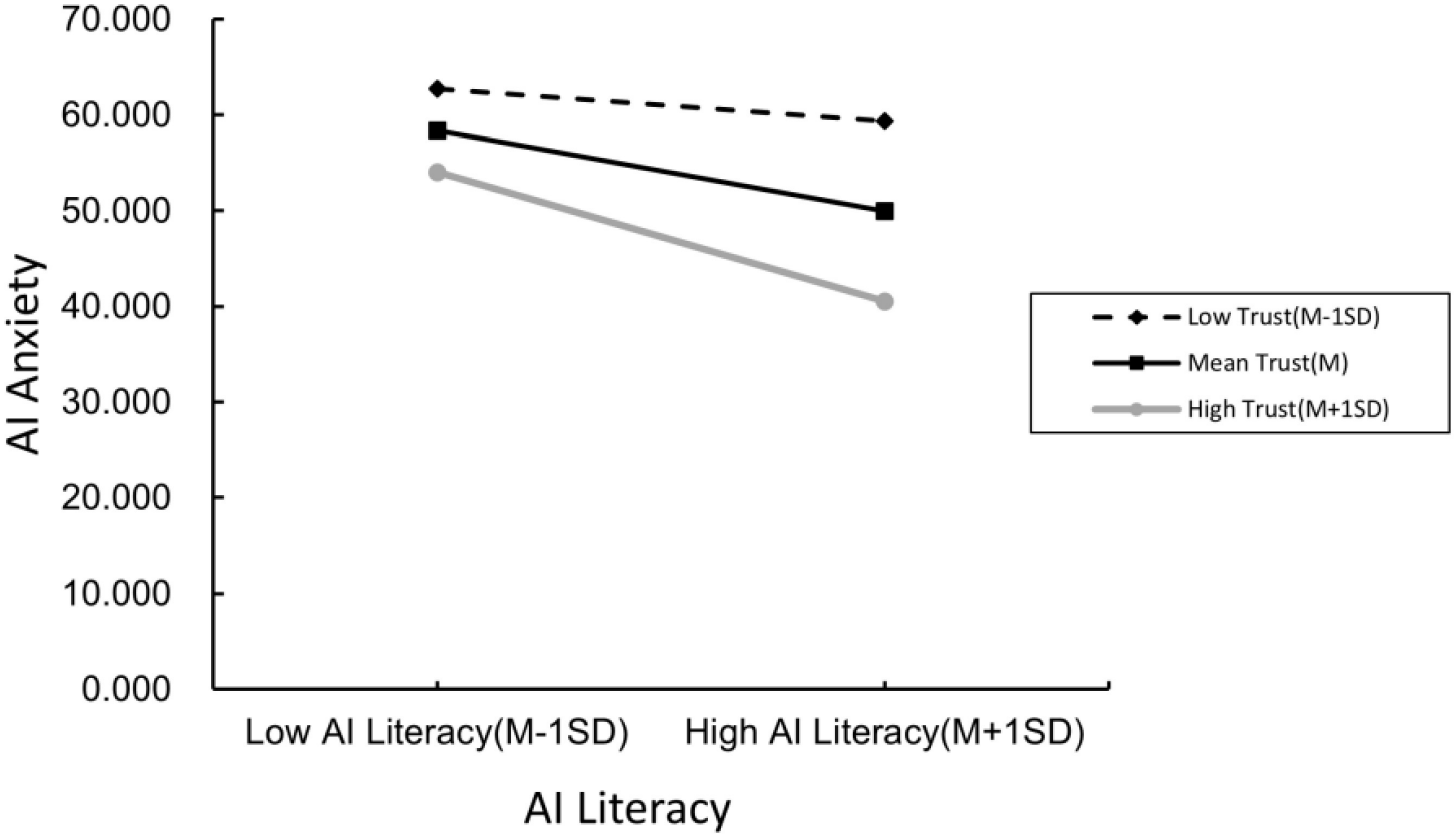
Simple slope plot.

To further understand the nature of the moderating effect, we employed Aiken and West’s (1991) method to conduct simple slope tests and plot simple effects analysis plots (Figure 2). These examined the predictive effect of AI literacy on AI anxiety was further examined at low levels of trust (-1 SD), mean levels of trust, and high levels of trust (+1 SD). Specifically, under low levels of trust (-1 SD), the negative predictive effect of AI literacy on AI anxiety was relatively weak. At mean levels of trust, this negative predictive effect became significantly stronger. Under high levels of trust (+1 SD), the negative predictive effect of AI literacy on AI anxiety was the strongest. The simple slope analysis further demonstrated that, as trust increased, the negative relationship between AI literacy and AI anxiety became progressively stronger. In other words, among individuals with higher levels of trust, AI literacy was more effective in reducing AI anxiety.

These findings further confirmed the moderating role of trust in the relationship between AI literacy and AI anxiety, indicating that trust strengthened the anxiety-reducing effect of AI literacy.

### Third-order interaction effect

To examine whether the moderating effect of trust on the “AI literacy—AI anxiety” pathway varies across disciplinary categories (natural sciences vs. humanities and social sciences), a hierarchical (block-entry) multiple regression analysis was conducted to test the three-way interaction among AI literacy, trust, and disciplinary category on AI anxiety. The results are presented in Table 7. In Model 1, the control variables (AI literacy, trust, discipline category) significantly predicted AI anxiety, explaining 38.8% of variance, *F* (3,559) = 117.94, *p* < 0.001. After adding second-order interaction terms in Model 2, the explanatory power increased to 41.7%, *ΔR*^2^ = 0.03, *F*(3,556) = 9.18, *p* < 0.001. Model 3, incorporating the third-order interaction term, did not show a significant increase in explanatory power (*ΔR*^2^ = 0.00, *F*(1,555) = 0.38, *p* = 0.54), indicating that the third-order interaction effect was not significant.

**TABLE 7:** Hierarchical (block-entry) multiple regression analysis.

Variables	Model 1		Model 2		Model 3	
	β	*t*	β	*t*	β	*t*
Constant		35.87***		36.98***		33.22***
Discipline^a^	–0.10	–2.87**	–0.09	–2.82**	–0.10	–2.75**
AI literacy	–0.22	–5.30***	–0.26	–2.07*	–0.28	–2.15*
Trust	–0.45	–10.80***	–0.38	–3.06**	–0.39	–3.11**
AI literacy × trust			–0.17	–4.94***	–0.23	–2.22*
AI literacy × discipline			0.01	0.04	0.02	0.14
Trust × discipline			–0.09	–0.75	–0.09	–0.69
AI Literacy × trust × discipline					0.06	0.62
Model fit indices						
*R* ^2^	0.39		0.42		0.42	
Adjusted *R*^2^	0.38		0.41		0.41	
*ΔR* ^2^			0.03		0.00	
*F*	117.94		66.15		56.69	
*ΔF*			9.18		0.38	

The professional category is a dummy variable (1 = Humanities and Social Sciences, 2 = Natural Sciences). The negative coefficient indicates that, compared to the “Humanities and Social Sciences” group (coded as 1), the “Natural Sciences” group (coded as 2) exhibits significantly lower levels of AI anxiety. *p < 0.05, **p < 0.01, ***p < 0.001.

Further examination of regression coefficients reveals that both AI literacy (β = –0.28, *p* = 0.03) and trust (β = –0.39, *p* = 0.002) significantly negatively predict AI anxiety. This indicates that higher AI literacy and higher levels of trust are both associated with lower AI anxiety. The second-order interaction between AI and trust (β = –0.23, *p* = 0.030) was significant, indicating that AI literacy exerts a stronger mitigating effect on AI anxiety under conditions of high trust. However, the three-way interaction (AI literacy × trust × discipline) did not reach significance (β = 0.06, *p* = 0.54), indicating that trust’s buffering effect on AI literacy’s reduction of AI anxiety was consistent across both discipline category.

## Discussion

This study, grounded in the Technology Acceptance Model (TAM) framework, constructed a moderated mediation model of “AI literacy AI anxiety—perceived usefulness” with university students from diverse disciplinary backgrounds as the research subjects. The findings suggest that AI literacy is positively associated with perceived usefulness, with this relationship being partially mediated by AI anxiety. This demonstrates that AI literacy exerts dual cognitive and affective influences on university students’ perceived usefulness of AI. This finding supports the perspective of AI literacy as a critical competency variable (Ng et al., 2021) and aligns with existing research concluding that “AI literacy enhances technological acceptance attitudes by alleviating AI anxiety” (Huo et al., 2023; Kang et al., 2023; Zhang et al., 2024; Li and Zhao, 2025). Moreover, trust significantly amplified the anxiety-reducing effect of AI literacy, validating the theoretical expectation that trust serves as a crucial psychological resource for underlying perceived usefulness (Gefen et al., 2003; McKnight et al., 2011).

### AI literacy and perceived usefulness: cognitive-affective dual pathways

The findings suggest the possible existence of a dual ical expectation that trust serves ersity this relationship being partially AI literacy not only positively predicts college students’ perceived usefulness (H1 holds), but also indirectly enhances perceived usefulness by reducing AI anxiety (H2, H3). This artially m-affective usefu-path model expands the explanatory boundaries of the classic TAM framework, addressing its historical neglect of emotional responses. Consistent with Mayer and Salovey’s (1995) cognitive-affective system theory, students’ levels of AI anxiety substantially influence their technology adoption process. This interpretation is also aligned with a recent cross-sectional study in higher education, which found that technology readiness and social influence were positively associated with the perceived usefulness and effectiveness of generative AI, while anxiety showed a negative association, reinforcing the role of cognitive preparedness and social context in shaping technology acceptance within TAM-based frameworks (Sallam et al., 2025b). Merely enhancing AI knowledge and skills is insufficient to ensure positive attitudes; concurrent emotional support and psychological interventions are essential. Therefore, AI education should integrate emotional regulation elements into curriculum designuld inas psychological counseling, learning support, and anxiety assessment mechanismseto achieve synergistic cognitive and emotional development, thereby more effectively boosting students’ overall acceptance of AI.

### The moderating role of trust and the mechanism of social psychological resources

Further analysis indicates that trust exerts a significant moderating effect on the “AI literacy—AI anxiety” pathway. Trust does not operate independently but functions as a potent psychological buffer resource, substantially amplifying the positive impact of AI literacy while mitigating AI anxiety. This implies that for students with high levels of trust, improvements in AI literacy translate more effectively into heightened psychological security and reduced anxiety; conversely, low trust diminishes the psychological advantages that high literacy would otherwise confer. This finding illuminates trust’s role as an “amplifier” in technology acceptance (Siau and Wang, 2018). In other words, trust serves as a prerequisite for technology acceptance (Gefen et al., 2003). Even when an individual’s AI literacy is insufficient, high levels of trust can mitigate anxiety, thereby sustaining positive perceptions of usefulness.

This finding enriches TAM theory from a socio-psychological perspective, indicating that AI adoption is influenced not only by rational judgments but also by the process of trust formation. Such a “trust-driven” AI education strategy provides emotional security beyond cognitive factors, thereby fostering more stable attitudes toward technology acceptance. Therefore, integrating trust-building into AI literacy education frameworks entails: (1) Developing differentiated AI literacy curricula: Humanities and social sciences courses should focus on “AI anxiety management + ethical cognition,” reducing technological apprehension through case discussions (e.g., AI ethical controversies, career transition pathways); natural sciences courses should emphasize “technology application + critical thinking,” enhancing recognition of AI limitations and algorithmic biases; (2) Integrate AI ethical literacy cultivation: Incorporate ethical issues such as algorithmic fairness, privacy protection, and responsibility delineation into all subject-specific AI courses. Utilize formats like “ethical dilemma simulations” and “AI ethics debates” to enhance students’ cognitive and judgmental capabilities regarding AI ethics; (3) Trust-building pedagogy: Foster functional and humanistic trust in AI through “transparent AI decision-making demonstrations (e.g., deconstructing recommendation algorithm logic),” small-scale AI tool trial feedback, and “positive case sharing”; (4) Promoting interdisciplinary AI collaborative projects: Organizing joint participation by natural sciences and humanities/social sciences students in AI application projects (e.g., designing AI solutions for societal challenges) facilitates cross-disciplinary knowledge exchange and reduces psychological differences, serving as a key strategy for optimizing educational outcomes.

### Disciplinary background differences and interdisciplinary consistency

Research findings indicate that disciplinary background significantly influences students’ initial levels of AI literacy, anxiety, and trust, consistent with existing research conclusions (Becher and Trowler, 2001; Neumann et al., 2002; Venkatesh and Davis, 2000; Stöhr et al., 2024). Students in natural sciences generally exhibit higher AI literacy, trust, and perceived usefulness, alongside lower anxiety levels. This correlates closely with their prolonged immersion in technology-intensive learning environments (Elshaer et al., 2024; Raman et al., 2024; Stöhr et al., 2024; Thongsri et al., 2020). Conversely, humanities and social sciences students exhibited relatively higher levels of AI anxiety., particularly concerning job displacement and technophilic, reflecting their disciplinary paradigms’ greater emphasis on technology’s social, ethical, and humanistic implications (Van Dijk, 2020). This divergence demonstrates how disciplinary cultures profoundly shape students’ attitudes toward technology, underscoring the necessity of discipline-specific pedagogy: humanities and social sciences students require context-specific approaches to alleviate anxiety, while natural sciences students can further enhance their technological application capabilities.

However, of greater theoretical value is the cross-disciplinary consistency in students’ psychological mechanisms: the mediating pathway through which AI literacy influences perceived usefulness via anxiety, alongside the moderating effect of trust on this pathway, remains stable across both groups. The non-significance of third-order interaction effects further validates this pattern’s universality, indicating the model’s consistent applicability across different academic disciplines. Fundamentally, trust—as a core socio-psychological resource (Hobfoll, 2002)—exhibits cross-situational and cross-group commonality in its buffering function against technological uncertainty. For both natural science and humanities/social sciences students, trust in AI fundamentally stems from foundational perceptions of technological reliability, explainability, and ethical compliance. This core psychological demand remains unaffected by disciplinary knowledge systems or differences in cognitive paradigms. Secondly, the universal characteristics of AI technology determine the generalizability of trust regulation: both student groups encounter AI technologies possessing disciplinary knowledge systemtonomy. Their sources of anxiety (such as doubts about functional reliability and privacy security concerns) share fundamental commonalities. Trust exerts a moderating effect by reducing this shared risk perception, thus not diverging based on disciplinary background. Moreover, the shared environment of university AI educationcurity concerns) share fundamental commonalities. Trust exerts a moderating effect by reducing this shared risk-disciplinary consistency of trust regulation effects. Both groups of students develop attitudes toward AI within analogous educational ecosystems, reinforcing the stability of core psychological mechanisms. Practically, university AI education should thus adopt both differentiated and universalized strategies. On the one hand, differentiated educational starting points and content emphases should be adopted for students from different disciplines. On the other hand, trust-building should become a universal objective for all students, forming a cross-disciplinary psychological support system.

### Limitations and prospects

This study expands the explanatory power of TAM in the AI era by integrating the “cognitive-affective” perspective with subject-specific background variables, providing a comprehensive framework for understanding the psychological mechanisms of human-AI interaction. While yielding findings of both theoretical and practical value, the research has certain limitations that warrant further exploration and extension in future studies.

First, the cross-sectional questionnaire design employed in this study limits the ability to infer causal relationships between variables. While statistical analysis revealed significant correlations, cross-sectional data cannot determine causal direction and may be subject to common method bias. Future research may adopt longitudinal tracking or experimental intervention designs and incorporate behavioral indicators (e.g., AI system usage records and task performance) to improve the robustness of the findings.

Second, the study’s sample primarily originates from Chinese universities, therefore, the applicability of the findings across different cultural backgrounds and educational systems requires further verification. Beyond cross-cultural validation, future research should also compare the applicability of the model across different educational contexts, such as vocational education institutions, higher vocational colleges, research-oriented universities, and non-higher-education settings (e.g., corporate training and continuing education), in order to further examine whether human–AI interaction mechanisms remain consistent across different learning ecologies.

Third, the current model incorporates only AI anxiety and trust as core variables, failing to fully capture the complexity of AI acceptance. Future research should integrate additional individual factors (e.g., technological innovativeness, self-efficacy, learning motivation) alongside contextual and mechanistic factors (e.g., teacher support, institutional policies, curriculum design) to construct a more comprehensive AI acceptance ecosystem model.

Fourth, this study did not include behavioral variables such as AI usage frequency or prior AI exposure experience. Individuals’ familiarity with AI technologies may further influence their AI anxiety, trust, and perceived usefulness. Therefore, future studies could incorporate variables such as AI usage experience, frequency of technological exposure, and digital competence into the model to more accurately identify the independent effect of AI literacy.

In addition, although this study found significant mean differences among students from different disciplinary backgrounds in AI literacy, AI anxiety, trust, and perceived usefulness, the results of the three-way interaction analysis indicated that the moderated mediation mechanism remained relatively stable across disciplinary groups. Accordingly, this study primarily revealed differences in variable levels associated with disciplinary background rather than differences in the underlying mechanisms. Future research may employ approaches such as multi-group Structural Equation Modeling (multi-group SEM) or Latent Profile Analysis (LPA) to further explore potential differences in psychological characteristic structures and AI acceptance pathways across disciplinary groups.

Furthermore, this study conceptualized AI as a holistic technological construct and did not distinguish among different AI modalities, such as generative AI, recommendation algorithms, and intelligent assessment systems. However, different types of AI may evoke varying levels of trust, anxiety, and perceived usefulness. Therefore, future research could further differentiate AI technology types and compare college students’ psychological responses and acceptance mechanisms across different AI application contexts, thereby enhancing the contextual applicability of the findings.

Finally, given the rapid advancement of AI education, future research may explore emerging variables such as AI ethical literacy and perceptions of algorithmic fairness. This would further enrich the applicability of the TAM model within intelligent education contexts, particularly by incorporating broader ethical dimensions such as data privacy, academic integrity, and equitable access, which may influence students’ trust, engagement, and effective use of AI technologies in educational settings (Sallam et al., 2025a).

## Conclusion

This study, grounded in the Technology Acceptance Model (TAM), tests a dual-path “cognitive-affective” moderated mediation model, which was supported by empirical data from a sample of 563 Chinese university students. Key findings include: (1) AI literacy positively mediates college students’ perceptions of AI usefulness; (2) AI anxiety mediates the relationship between AI literacy and perceived usefulness; (3) Trust exerts a protective moderating effect in the “AI literacy → AI anxiety” pathway, with high levels of trust mitigating anxiety stemming from low AI literacy. (4) Significant differences exist in AI literacy, AI anxiety, trust, and perceived usefulness between humanities/social sciences and natural sciences students. However, third-order interaction results indicate that trust’s moderating effect is consistent across disciplinary groups.

## Research significance

### Theoretical significance

Grounded in the Technology Acceptance Model (TAM), this study examined the psychological mechanism through which AI literacy influences college students’ perceived usefulness of AI from a cognitiveefulness of AI sychological mechanism through which AI literacy influences collas well as the moderating role of trust. The findings indicate that college students’ perceived usefulness of AI is not only shaped by cognitive ability factors, but is also jointly influenced by emotional states and socio-psychological resources, thereby extending the explanatory boundaries of TAM in intelligent education contexts.

In addition, this study further found that the moderated mediation mechanism remained relatively stable across different disciplinary backgrounds, suggesting the existence of a potentially cross-disciplinary general psychological mechanism in the formation of perceived usefulness of AI. Compared with prior research that has mainly focused on technological characteristics or single psychological variables, this study integrates AI literacy, AI anxiety, and trust within a unified framework, providing a new theoretical perspective for understanding the psychological mechanisms underlying human–AI interaction.

### Practical significance

The findings of this study may provide useful implications for AI education and curriculum design in higher education institutions. On one hand, in the process of promoting AI education, universities should not only emphasize the development of students’ AI knowledge and skills, but also pay attention to their emotional experiences and psychological adaptation in AI use. Efforts should be made to help students reduce AI anxiety and develop positive attitudes toward technology. On the other hand, the moderating effect of trust suggests educators should enhance students’ trust in AI systems through transparent tool design, interpretable results presentation, and dissemination of positive case studies, thereby mitigating anxiety stemming from low literacy. Finally, universities should design differentiated teaching approaches based on disciplinary backgrounds: students in humanities and social sciences should focus on reducing anxiety and enhancing ethical awareness, while those in natural sciences should emphasize reflecting on the societal impacts of AI applications.

In summary, the empirical findings of this study provide concrete references for constructing university AI curriculum systems, designing psychological support mechanisms, and formulating interdisciplinary educational policies. They also offer psychological insights for promoting students’ healthy adaptation to the AI era.

## Data Availability

The datasets presented in this study can be found in online repositories. The names of the repository/repositories and accession number(s) can be found in the article/supplementary material.
